# FAM136A depletion induces mitochondrial stress and reduces mitochondrial membrane potential and ATP production

**DOI:** 10.1002/2211-5463.13967

**Published:** 2025-01-16

**Authors:** Yushi Otsuka, Masato Yano

**Affiliations:** ^1^ Department of Medical Technology, Faculty of Health Sciences Kumamoto Health Science University Kumamoto Japan

**Keywords:** ATP, FAM136A, holocytochrome c synthetase, mitochondrial membrane potential, mitochondrial stress

## Abstract

FAM136A deficiency has been associated with Ménière's disease. However, the underlying mechanism of action of this protein remains unclear. We hypothesized that FAM136A functions in maintaining mitochondria, even in HepG2 cells. To better characterize FAM136A function, we analyzed the cellular response caused by its depletion. FAM136A depletion induced reactive oxygen species (ROS) and reduced both mitochondrial membrane potential and ATP production. However, cleaved caspase‐9 levels did not increase significantly. We next investigated why the depletion of FAM136A reduced the mitochondrial membrane potential and ATP production but did not lead to apoptosis. Depletion of FAM136A induced the mitochondrial unfolded protein response (UPR^mt^) and the expression levels of gluconeogenic phosphoenolpyruvate carboxykinases (PCK1 and PCK2) and ketogenic 3‐hydroxy‐3‐methylglutaryl‐CoA synthases (HMGCS1 and HMGCS2) were upregulated. Furthermore, depletion of FAM136A reduced accumulation of holocytochrome c synthase (HCCS), a FAM136A interacting enzyme that combines heme to apocytochrome c to produce holocytochrome c. Notably, the amount of heme in cytochrome c did not change significantly with FAM136A depletion, although the amount of total cytochrome c protein increased significantly. This observation suggests that greater amounts of cytochrome c remain unbound to heme in FAM136A‐depleted cells.

## Graphical abstract

FAM136A depletion upregulated ROS production, reduced mitochondrial membrane potential (ΔΨ) and ATP production, and upregulated expression of PCK1, PCK2, HMGCS1, and HMGCS2. The expression of both TOMM22 and TOMM20 was also upregulated. FAM136A depletion reduced HCCS that produce holocytochrome c by combining heme to apocytochrome c, and reduced the ratio of holocytochrome c/apocytochrome c.

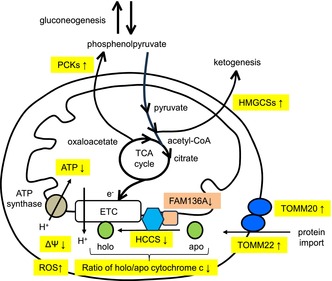

**Abbreviations**
2‐ME: 2‐mercaptoethanol
ATF4 and ATF5: activating transcription factor 4 and 5
ATP5A: mitochondrial ATP synthase subunit alpha
CASP9: caspase‐9
CS: citrate synthase
DDIT3: DNA damage‐inducible transcript 3 (CHOP)
ECL: enhanced chemiluminescence
ETC: electron transport chain
FAM136A: family with sequence similarity 136 member A
GAPDH: glyceraldehyde‐3‐phosphate dehydrogenase
HBSS: Hank's balanced salt solution
HCCS: holocytochrome c synthase
HMGCS1 and HMGCS2: 3‐hydroxy‐3‐methylglutaryl‐CoA synthase 1 and 2
HSPD1: heat shock protein family D (Hsp60) member 1
HTRA2: high‐temperature requirement protein A2
IM: inner membrane
IMS: intermembrane space
ISR: integrated stress response
LONP1: Lon peptidase 1
MTCO1: mitochondrially encoded cytochrome *c* oxidase I
OM: outer membrane
PCK1 and PCK2: phosphoenolpyruvate carboxykinase 1 and 2
PVDF: polyvinylidene difluoride
siRNA: small interfering RNA
TOM: translocase of mitochondrial outer membrane
TOMM20, TOMM22, and TOMM40: translocase of outer mitochondrial membrane 20, 22, and 40
UPR^mt^
: mitochondrial unfolded protein response
UQCRC2: ubiquinol‐cytochrome *c* reductase core protein 2
VDAC1: voltage‐dependent anion channel 1

Mitochondria are organelles with two distinct membranes (the outer and inner membrane [OM and IM, respectively]) that divide the organelle into two soluble compartments and include the intermembrane space (IMS) and mitochondrial matrix. Most mitochondrial proteins are encoded by nuclear genomic DNA. mRNAs encoding mitochondrial proteins are translated into proteins on cytosolic ribosomes and imported into the mitochondria [1, 2, 3, 4]. When nuclear‐encoded mitochondrial proteins are imported into the mitochondrial matrix, most are recognized by receptor proteins, such as TOMM22 and TOMM20, in the translocase of the mitochondrial outer membrane (TOM) complex [5]. The mitochondrial proteins are then sorted into the IMS across the pore formation protein—TOMM40. Subsequently, they move into the mitochondrial matrix with the help of the translocase of the mitochondrial inner membrane complex.

Mitochondria perform many functions, including ATP generation, metabolism, and apoptosis. ATP generation plays a vital role in mitochondria. The electron transport chain (ETC) coupled with ATP synthase consumes oxygen [6, 7]. However, the ETC cannot transport electrons with absolute efficiency, and the electrons leak to some extent and react with oxygen to form reactive oxygen species (ROS) [8]. ROS tend to react with and modify DNA, unsaturated lipids, and proteins [9]. Therefore, ROS generated primarily in the mitochondria tend to damage mitochondrial DNA, membrane lipids, and proteins [10].

The mitochondrial unfolded protein response (UPR^mt^) is activated when damaged or misfolded proteins accumulate in the mitochondria [11, 12, 13]. In humans, mitochondrial stress signals upregulate transcription factors, such as ATF4, ATF5, and DDIT3 [14]. These enhance UPR^mt^ by inducing several enzymes, including HSPD1 (a mitochondrial matrix molecular chaperone that folds newly imported mitochondrial proteins) and LONP1 (a mitochondrial matrix protease that digests aggregated mitochondrial proteins). Furthermore, mitochondrial protein import is enhanced when UPR^mt^ is induced [15]. Indeed, the increase in the protein expression levels of the mitochondrial import receptors—TOMM22 and TOMM20—during the UPR^mt^ has been reported [16, 17].

FAM136A is an orphan protein that localizes to the mitochondrial IMS [18]. The *FAM136A* gene has been associated with Ménière's disease [19]. A recent analysis using *Caenorhabditis elegans* demonstrated that the loss of FAM136A function resulted in subtle but significant changes in locomotion and behavior [20]. Another recent analysis indicated that FAM136A null mice exhibit hearing deficiency [21]. However, to the best of our knowledge, no other functional information on this protein has been reported.

We hypothesized that FAM136A plays a fundamental role in mitochondrial maintenance in all cell types, including HepG2 cells. We analyzed the cellular responses induced by FAM136A depletion in HepG2 cells. FAM136A depletion increased ROS production and decreased mitochondrial membrane potential and ATP production, but no significant increase in cleaved caspase‐9 (CASP9) was observed, suggesting that an unknown cell response may inhibit apoptosis. Upregulation of UPR^mt^‐related proteins (ATF4, DDIT3, and LONP1) and enhanced expression of mitochondrial import receptors (TOMM22 and TOMM20) were also observed. We hypothesized that gluconeogenic phosphoenolpyruvate carboxykinases (PCK1 and PCK2) and ketogenic 3‐hydroxy‐3‐methylglutaryl‐CoA synthases (HMGCS1 and HMGCS2) may prevent ROS overproduction by downregulating tricarboxylic acid (TCA) cycle in the mitochondria. Therefore, whether the expression levels of these four enzymes were regulated by FAM136A depletion was investigated. Thereafter, we examined whether these four enzymes have a function to prevent ROS generation in FAM136A‐depleted cells, because it has been recently reported that PCK1 has a function to suppresses ROS generation, as was [22]. In addition, recent interactome analysis has experimentally indicated that FAM136A interacts with holocytochrome c synthase (HCCS), an enzyme that combines heme with apocytochrome c [23]. Therefore, we further examined the expression level of HCCS and cytochrome c, and heme content.

## Materials and methods

### Reagents and antibodies

Unless otherwise stated, all reagents were purchased from Sigma‐Aldrich (St. Louis, MO, USA), Wako (Osaka, Japan), or Takara (Kyoto, Japan). The anti‐DYKDDDDK (FLAG) antibody used for immunostaining was purchased from Wako. Anti‐FAM136A, HTRA2, ATF4, ATF5, DDIT3, CASP9, PCK1, PCK2, HMGCS1, and HCCS antibodies were purchased from Proteintech (Rosemont, IL, USA). Anti‐cytochrome c antibody was purchased from R&D Systems (Minneapolis, MN, USA). Anti‐HSPD1 and anti‐LONP1 antibodies were purchased from Sigma‐Aldrich. Anti‐VDAC1 (Calbiochem‐Novabiochem, San Diego, CA, USA), anti‐GAPDH (Novus Biologicals, CO, USA), anti‐citrate synthase (CS) (Nordic‐MUbio, Susteren, the Netherlands), anti‐HMGCS2 antibody (Abcam, Cambridge, UK), and Total OXPHOS Rodent WB Antibody Cocktail (Abcam) containing anti‐ATP5A, UQCRC2, and MTCO1 antibodies were also purchased. Anti‐TOMM20, TOMM22, and TOMM40 antibodies were obtained as previously described [24, 25, 26]. The plasmid pCMV6‐ FAM136A‐Myc‐FLAG used to express human FAM136A tagged with Myc and FLAG sequences at the C terminus was purchased from OriGene (Rockville, MD, USA). The plasmids pPCK1 (pRP[Exp]‐CAG > hPCK1[NM_002591.4]), pPCK2 (pRP[Exp]‐CAG > hPCK2[NM_004563.4]), pHMGCS1 (pRP[Exp]‐Neo‐CAG > hHMGCS1[NM_001330663_2]), and pHMGCS2 (pRP[Exp]‐Neo‐CAG > hHMGCS2[NM 005518_4]) used to express human PCK1, PCK2, HMGCS1, and HMGCS2 were obtained from VectorBuilder (Chicago, IL, USA). The blank plasmid pControl (pRP[Exp]‐CAG > ORF_Stuffert) was also obtained from VectorBuilder. HepG2 cells (Cell No. RCB1648) were obtained from RIKEN BioResource Research Center (Ibaraki, Japan).

### Immunostaining FAM136A‐Myc‐FLAG‐expressing cells

HepG2 cells cultured on coverslips were transfected with pCMV6‐FAM136A‐Myc‐FLAG using Lipofectamine 3000 (Invitrogen, Carlsbad, CA, USA) then stained with MitoTracker Red and fixed with 4% paraformaldehyde. The cells were treated with phosphate‐buffered saline (PBS) containing 1% Triton X‐100 and then with an anti‐FLAG antibody and goat anti‐mouse IgG conjugated with Alexa Fluor488 (Abcam). The cells were stained with Hoechst dye (Sigma‐Aldrich). Fluorescence of Alexa Fluor488 (green), MitoTracker Red (red), and Hoechst dye (blue) were imaged using a fluorescence microscope.

### Alkali‐, digitonin‐, hypotonic extraction assays and isolation of mitochondria

HepG2 cells were harvested in ice‐cold PBS containing 1 mM EDTA then washed with PBS. They were suspended in the isolation buffer (3 mm HEPES‐KOH [pH 7.4], 0.21 m mannitol, 0.07 m sucrose, and 0.2 mm EGTA), and homogenized on ice using a Dounce homogenizer (Wheaton, IL, USA). The homogenate was centrifuged at 500 ***g***, and the supernatant containing the mitochondria was collected. The supernatant was further centrifuged at 10 000 ***g*** and the mitochondrial fraction was collected as a pellet. The isolated mitochondria were sonicated and extracted with 0.1 m Na_2_CO_3_ (pH 11.5) for the alkali extraction assay using bicarbonate, as described previously [25]. Following centrifugation at 100 000 ***g***, the supernatant was collected as the mitochondrial soluble fraction, and the pellet was collected as the mitochondrial membrane fraction. We performed a digitonin extraction assay to analyze the localization of mitochondria‐soluble proteins in the IMS or matrix. The isolated mitochondria were treated with an isolation buffer containing digitonin at 0.1, 0.2, 0.4, and 0.8 mg·mL^−1^ and centrifuged at 10 000 ***g***. Supernatants and pellets from each sample were mixed with sodium dodecyl sulphate/polyacrylamide gel electrophoresis (SDS/PAGE) loading buffer for immunoblot analysis. We performed hypotonic extraction assay to confirm FAM136A localization in IMS. Isolated mitochondria were treated with the hypotonic buffer (3 mm HEPES‐KOH [pH 7.4], 10 mm sucrose, and 0.2 mm EGTA) and centrifuged at 10 000 ***g***. The pellets were further suspended with PBS and then centrifuged again to obtain supernatants and pellets.

### Transfection of small interfering RNAs and plasmids into HepG2 cells

Lipofectamine RNAiMAX (Invitrogen) and Opti‐MEM I (Gibco, New York, NY, USA) were used to transfect siRNAs into HepG2 cells. The universal negative control small interfering RNAs (siRNA) and siRNAs used to knockdown FAM136A were purchased from JBioS (Saitama, Japan). The sequence of negative control siRNA and FAM136A‐targeted siRNAs No.1–5 are shown in Table 1. After transfection with the siRNAs, the cells were cultured for 72 h for subsequent analysis. The transfection of siRNAs and plasmids was performed using Lipofectamine 3000 (Invitrogen).

**Table 1:** siRNAs used in this study.

	Sense	Antisense
Negative control siRNA	5′‐UUCUCCGAACGUGUCACGUdTdT‐3′	5′‐ACGUGACACGUUCGGAGAAdTdT‐3′
FAM136A‐targeted siRNA No.1	5′‐CGACAAAGCCAAAGAUUCAdTdT‐3′	5′‐UGAAUCUUUGGCUUUGUCGdTdT‐3′
FAM136A‐targeted siRNA No.2	5′‐GUCUUUUAAGCAAAGUUUAdTdT‐3′	5′‐UAAACUUUGCUUAAAAGACdTdA‐3′
FAM136A‐targeted siRNA No.3	5′‐GGUACCAGACUCUUCUUACdTdT‐3′	5′‐GUAAGAAGAGUCUGGUACCdTdT‐3′
FAM136A‐targeted siRNA No.4	5′‐GGUCACUAACUUAGAAGGGdTdT‐3′	5′‐CCCUUCUAAGUUAGUGACCdTdC‐3′
FAM136A‐targeted siRNA No.5	5′‐GGUAGACUAGUUUGGAUAAdTdT‐3′	5′‐UUAUCCAAACUAGUCUACCdTdC‐3′

### Immunoblot analysis

HepG2 cells were collected, washed, and lysed in PBS containing 1% Triton X‐100 for the immunoblot analysis. After centrifugation at 10 000 ***g***, the supernatant was mixed with the SDS/PAGE loading buffer. After SDS/PAGE, the proteins were transferred onto polyvinylidene difluoride (PVDF) membranes. Membranes were blocked with 5% skim milk and subjected to immunoblot analysis using an enhanced chemiluminescence (ECL) western blotting detection reagent (GE Healthcare, Buckinghamshire, UK). Immunoblotting was performed using the abovementioned antibodies. Chemiluminescent images of the membranes were obtained using Ez‐Capture MG (ATTO, Tokyo, Japan). The intensities (protein expression levels) of the detected bands were analyzed using the imagej software (https://imagej.net/ij/).

### Quantitative polymerase chain reaction

Total RNA was isolated from HepG2 cells using TRIzol reagent (Invitrogen). The isolated RNA was reverse‐transcribed into cDNA using the PrimeScript RT Reagent Kit (Takara). Quantitative polymerase chain reaction (qPCR) was performed using cDNA as the template on a LightCycler Nano System (Roche). The oligonucleotides used for qPCR are listed in Table 2. Target mRNA expression levels were normalized to GAPDH mRNA expression levels.

**Table 2:** Primers used for quantitative PCR.

Target gene	Forward primer	Reverse primer
GAPDH	5′‐TGACAACAGCCTCAAGAT‐3′	5′‐GAGTCCTTCCACGATACC‐3′
FAM136A	5′‐TGCAGGGTCTCATGTTCCG‐3′	5′‐GCTCCTTACTCCCAGCATCTATT‐3′
PCK1	5′‐AAAACGGCCTGAACCTCTCG‐3′	5′‐ACACAGCTCAGCGTTATTCTC‐3′
PCK2	5′‐GCCATCATGCCGTAGCATC‐3′	5′‐AGCCTCAGTTCCATCACAGAT‐3′
HMGCS1	5′‐GATGTGGGAATTGTTGCCCTT‐3′	5′‐ATTGTCTCTGTTCCAACTTCCAG‐3′
HMGCS2	5′‐GACTCCAGTGAAGCGCATTCT‐3′	5′‐CTGGGAAGTAGACCTCCAGG‐3′

### Measurement of ROS generation, mitochondrial membrane potential, and ATP content

HepG2 cells were cultured in microwell plates, as described above. After siRNA transfection, the cells were cultured for 72 h and used to quantify ROS generation, detect mitochondrial membrane potential, and measure ATP content. To quantify ROS generation, the cells were treated with CM‐H_2_DCFDA (Invitrogen) and washed three times with Hank's balanced salt solution (HBSS). The intensity of green fluorescence (excitation, 485 nm; emission, 535 nm) was measured using Infiniti F200 Pro (TECAN, Zürich, Switzerland). To detect mitochondrial membrane potential, the cells were treated with JC‐1 (Dojindo, Kumamoto, Japan) then washed with HBSS. The intensities of green (excitation, 485 nm; emission, 535 nm) and red (excitation, 535 nm; emission, 590 nm) fluorescence were measured using Infiniti F200 Pro. The cells were harvested and lysed in PBS containing 1% Triton X‐100 to measure ATP content. After centrifugation, the concentrations of ATP and proteins in the supernatant were determined using an ATP detection kit (Dojindo, Kumamoto, Japan). The protein concentration in the supernatant was measured using a Bradford Protein Assay Kit (Takara).

### Detection of heme in cytochrome c

To detect heme bound to cytochrome c, HepG2 cells were collected, washed, and lysed in PBS containing 1% Triton X‐100, and centrifuged at 10 000 ***g***. The supernatant was mixed with SDS/PAGE loading buffer without a reducing agent, 2‐mercaptoethanol (2‐ME), to maintain the covalent bonds between the heme and cytochrome c protein. The proteins were then transferred onto PVDF membranes. After washing with PBS, heme in proteins was detected using an ECL kit as described previously [27]. After detection of heme, the same membrane was subjected to immunoblot analysis using anti‐cytochrome c antibody to confirm that the detected heme signal corresponded to the heme in holocytochrome c.

### Morphological analysis of mitochondria

HepG2 cells cultured on coverslips were transfected with negative control siRNA and FAM136A‐targeted siRNA No.1 using Lipofectamine RNAiMAX (Invitrogen) and Opti‐MEM I. The cells were then stained with MitoTracker Red, fixed with 4% paraformaldehyde, washed with PBS, and stained with Hoechst dye. Fluorescences of MitoTracker Red (red) and Hoechst dye (blue) were imaged using a fluorescence microscope.

### Statistical analysis

Data were analyzed using a Student's *t*‐test and reported as mean ± standard deviation (SD). Data were assumed to be normally distributed, but this was not formally tested.

## Results

### Confirmation of the localization of FAM136A in mitochondrial IMS

**Fig. 1:**
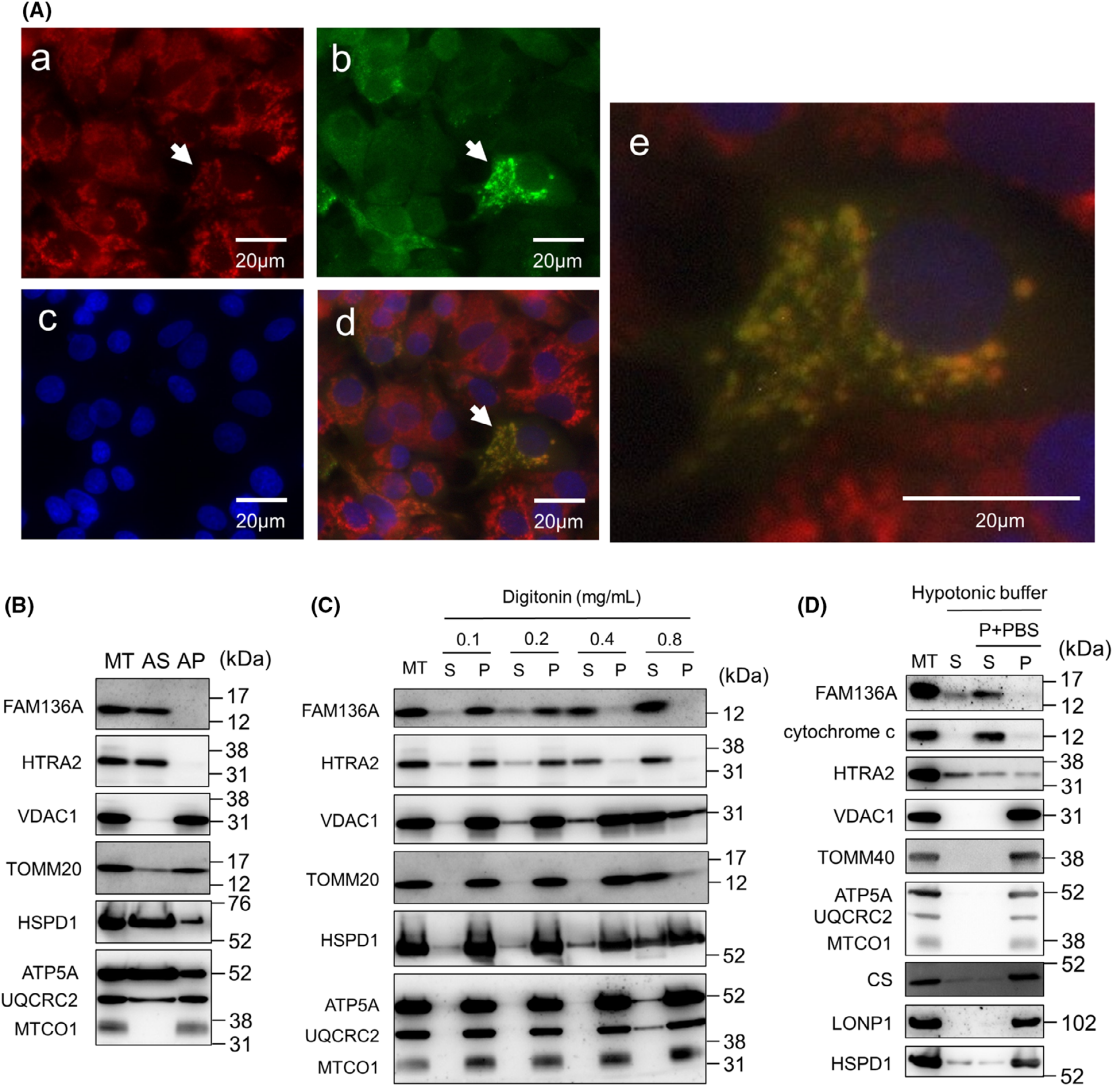
Confirmation of mitochondrial IMS localization of FAM136A. (A) HepG2 cells transfected with the vector expressing FAM136A‐Myc‐FLAG were stained with Mito Tracker Red (a), anti‐FLAG antibody and secondary antibody conjugated with Alexa Fluor488 (b), and Hoechst (c); the merged images of these three photographs are also shown (d), and the cell image with arrow shown in (d) was enlarged (e). (B) Mitochondria were isolated from HepG2 cells, as described in the Materials and Methods section, and subjected to an alkali extraction assay. Then, immunoblot analysis was performed. (C) Mitochondria isolated from HepG2 cells were treated with digitonin at the indicated concentration, followed by centrifugation to obtain the supernatant and pellet fractions (S, supernatant fraction; P, pellet fraction). (D) Mitochondria isolated from HepG2 cells were treated with hypotonic solution followed by centrifugation to obtain the supernatant fraction (S). The remained pellet suspended by PBS (P + PBS) was then centrifuged to obtain the supernatant (S) and pellet (P) fraction. AP, alkali‐resistant pellet fraction; AS, alkali‐soluble supernatant fraction; ATP5A, mitochondrial ATP synthase subunit alpha; HSPD1, heat shock protein family D (Hsp60) member 1; HTRA2, high‐temperature requirement protein A2; MT, isolated mitochondria; MTCO1, mitochondrially encoded cytochrome c oxidase I; TOMM20, translocase of outer mitochondrial membrane 20; UQCRC2, ubiquinol‐cytochrome *c* reductase core protein 2; VDAC1, voltage dependent anion channel 1.

The HepG2 cells transfected with pCMV6‐FAM136A‐Myc‐FLAG expressing FAM136A‐Myc‐FLAG were treated with MitoTracker Red, immunostained with an anti‐FLAG antibody and anti‐mouse IgG conjugated with Alexa Fluor488, and stained with Hoechst dye (Fig. 1A). The fluorescence image of Alexa Fluor488, indicated by the “arrow,” was almost identical to the fluorescence image of MitoTracker Red. These results indicate that FAM136A localizes to the mitochondria. Next, we investigated the localization region of FAM136A in the mitochondria. Mitochondria isolated from HepG2 cells were subjected to an alkali (bicarbonate) extraction assay using 0.1 m Na_2_CO_3_ (pH 11.5) (Fig. 1B). FAM136A was detected in the alkali‐soluble supernatant fraction, as well as HTRA2 (high‐temperature requirement protein A2, residing in IMS), HSPD1 (Hsp60, residing in the matrix), and ATP5A (ATP synthase subunit alpha, associated with IM). However, VDAC1 (voltage‐dependent anion channel 1, embedded in OM), TOMM20 (translocase of the outer membrane anchored to OM by a single transmembrane region), and MTCO1 (mitochondrially encoded cytochrome c oxidase I, embedded in IM) were detected in the alkali‐insoluble pellet fraction. These results indicate that FAM136A is a mitochondrial‐soluble protein that resides in either the IMS or the matrix. We performed a digitonin extraction assay using isolated mitochondria (Fig. 1C). When mitochondria were treated with 0.1 mg·mL^−1^ digitonin, all the proteins tested were mainly detected in the pellet. When mitochondria were treated with 0.4 mg·mL^−1^ digitonin, the majority of FAM136A was observed in the supernatant, and the majority of HTRA2 that resides in the IMS was also observed in the supernatant, whereas the majority of both VDAC1 and TOMM20 localized in the OM were observed in the pellet. Most other proteins localized in the matrix (HSPD1) or IM (ATP5A and MTCO1) were also detected in the pellet. In addition, isolated mitochondria were treated with hypotonic buffer (Fig. 1D). In this assay, the pellet fraction obtained by hypotonic buffer treatment followed by centrifugation was resuspended in PBS to restore isotonicity and further centrifuged to obtain supernatant and pellet. As a result, the majority of the FAM136A protein was found in the same fraction as cytochrome c. Therefore, FAM136A was indicated to be a mitochondrial IMS protein that probably interacts with the membrane.

### FAM136A depletion upregulates ROS production and reduces mitochondrial membrane potential and ATP production but does not induce obvious apoptosis

**Fig. 2:**
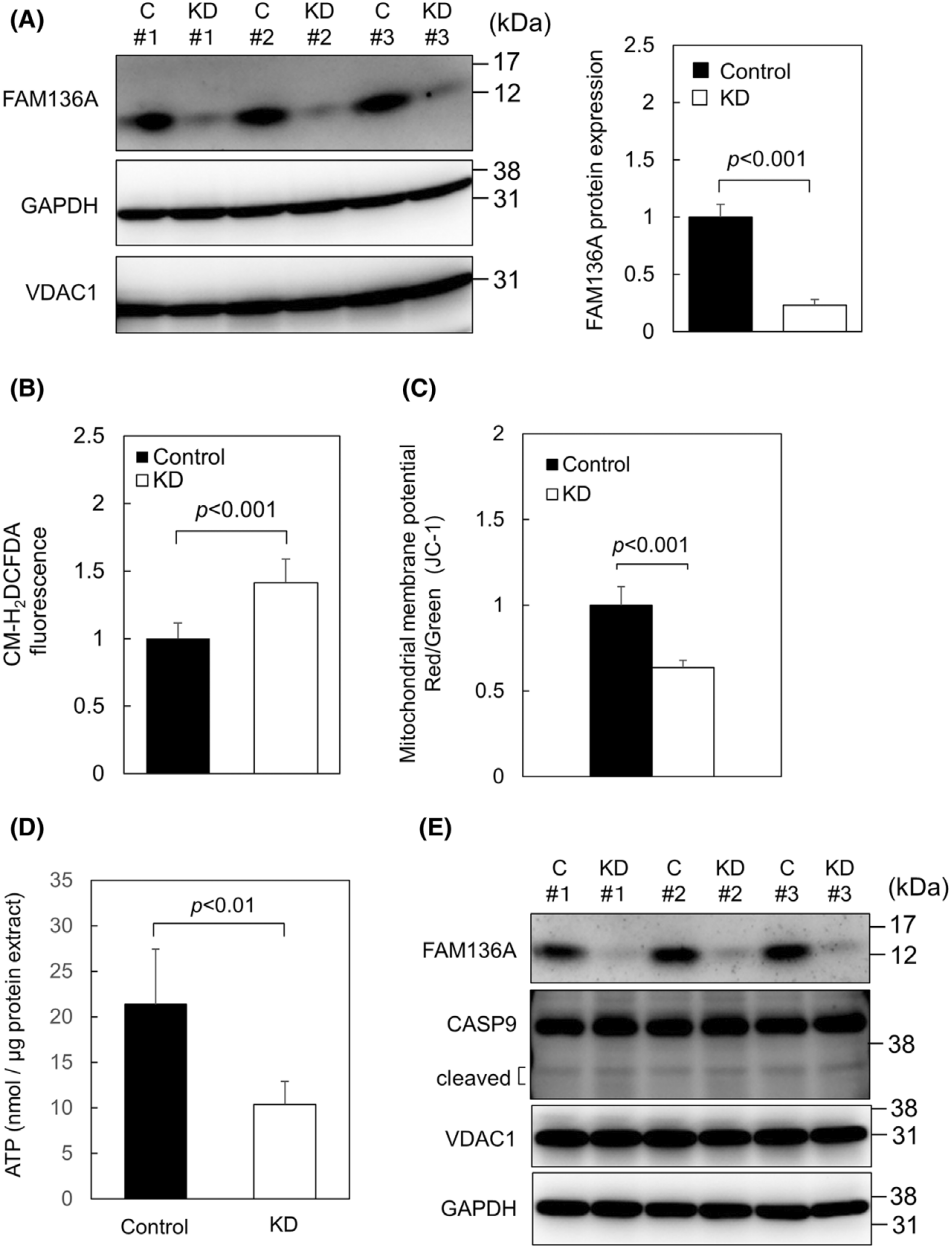
Depletion of FAM136A upregulates ROS production and reduces mitochondrial membrane potential and ATP production but does not induce obvious apoptosis. HepG2 cells were transfected with negative control siRNA or FAM136A‐targeted siRNA No. 1 and cultured for 72 h. (A) Proteins were extracted from the cells and subjected to immunoblot analysis to determine the FAM136A protein levels (*n* = 3). Protein expression of FAM136A and GAPDH was quantified, and normalized by GAPDH expression level. The protein expression levels of the control were set to 1. Data were analyzed using a Student's *t*‐test. Bars and whiskers of the graph represent mean and SD (right panel). (B) Fluorescence intensity of CM‐H_2_DCFDA‐loaded cells was measured using Infiniti F200 Pro (*n* = 8). The levels of CM‐H_2_DCFDA fluorescence in the control were set to 1. Data were analyzed using a Student's *t*‐test. Bars and whiskers of the graph represent mean and SD. (C) Fluorescence intensity of JC‐1‐loaded cells was measured using Infiniti F200 Pro (*n* = 32). The ratio of red fluorescence to green fluorescence (Red/Green) was calculated. The ratio of the control was set to 1. Data were analyzed using a Student's *t*‐test. Bars and whiskers of the graph represent mean and SD. (D) Intracelllular ATP concentration was quantified, and normalized by protein concentration (*n* = 4). Data were analyzed using a Student's *t*‐test. Bars and whiskers of the graph represent mean and SD. (E) Proteins extracted from the cells were subjected to immunoblot analysis to detect cleaved CASP9. CASP9, caspase‐9. C, control cell lysate; GAPDH, glyceraldehyde‐3‐phosphate dehydrogenase; KD, FAM136A‐depleted cell lysate obtained by using FAM136A‐targeted siRNA No.1.

To observe HepG2 cellular damage caused by FAM136A knockdown, HepG2 cells were transfected with negative control siRNA and FAM136A‐targeted siRNA No.1. After 72 h, the protein expression levels were examined (Fig. 2A). We were able to confirm that FAM136A‐targeted siRNA No.1 depleted FAM136A sufficiently. We next quantified the ROS levels in the cells using CM‐H_2_DCFDA because it is possible that a lack of FAM136A damages mitochondria (Fig. 2B). A significant increase in CM‐H_2_DCFDA fluorescence (green fluorescence) was detected in FAM136A‐lacking cells, indicating that the lack of FAM136A led to increased ROS generation. To analyze the mitochondrial membrane potential in FAM136A‐depleted cells, we used the fluorescent dye JC‐1. JC‐1 shows red fluorescence when the mitochondrial membrane potential is high and green fluorescence when the potential is low. A decrease in the ratio of red fluorescence to green fluorescence (red/green) was observed (Fig. 2C), indicating that the mitochondrial membrane potential was reduced in FAM136A‐depleted cells. In addition, we analyzed the influence of FAM136A depletion on ATP content (Fig. 2D). As a result, ATP level in FAM136A‐depleted cells markedly decreased, suggesting that ATP production was inhibited. However, we did not observe a significant increase in cleaved CASP9 expression using immunoblotting (Fig. 2E). Therefore, we hypothesized that an unknown cellular response in FAM136A‐depleted cells inhibits apoptosis signal transduction. We next analyzed possible cellular responses to reveal why the depletion of FAM136A reduced the mitochondrial membrane potential and ATP production but did not lead to apoptosis.

### FAM136A depletion induces ATF4‐ and DDIT3‐dependent UPR^mt^

**Fig. 3:**
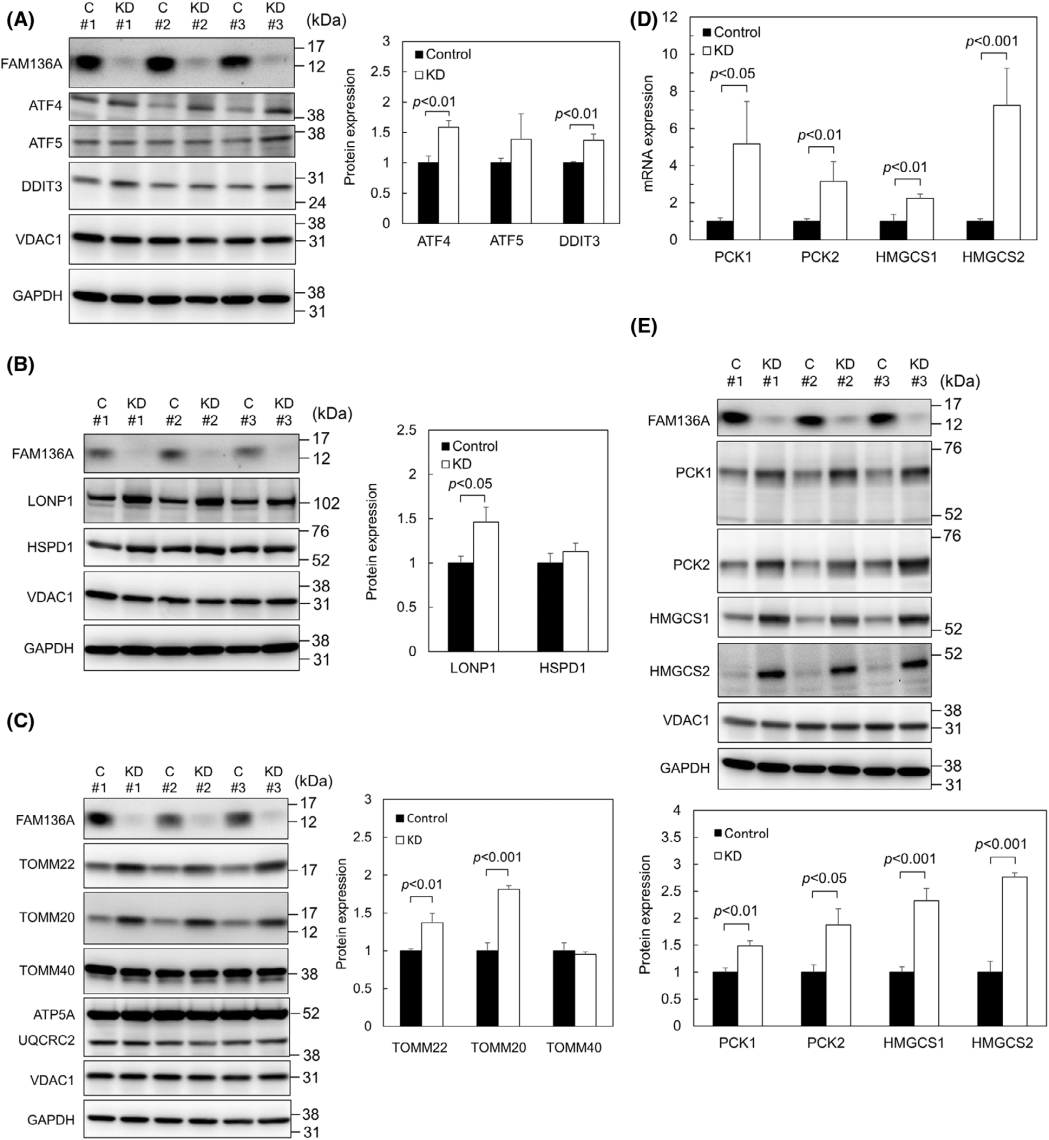
FAM136A depletion induced ATF4‐ and DDIT3‐dependent UPR^mt^, including upregulation of LONP1, TOMM22, TOMM20, PCK1, PCK2, HMGCS1, and HMGCS2. HepG2 cells were transfected with negative control siRNA or FAM136A‐targeted siRNA No.1 and cultured for 72 h. (A) Proteins extracted from the cells were subjected to immunoblot analysis to determine ATF4, ATF5, and DDIT3 protein levels (*n* = 3). ATF4 and ATF5, activating transcription factor 4 and 5; C, control cell lysate; DDIT3, DNA damage‐inducible transcript 3 (CHOP) (left panel); KD, FAM136A‐depleted cell lysate obtained by using FAM136A‐targeted siRNA No.1. Protein expression of ATF4, ATF5, DDIT3, and GAPDH was quantified, and normalized by GAPDH expression level. The protein expression levels of the control were set to 1. Data were analyzed using a Student's *t*‐test. Bars and whiskers of the graph represent mean and SD. (right panel) (B) Immunoblot analysis was performed to determine LONP1 and HSPD1 protein levels (*n* = 3). HSPD1, heat shock protein family D (Hsp60) member 1; LONP1, Lon peptidase 1 (left panel). Protein expressions of LONP1, HSPD1, and GAPDH were quantified, and normalized by GAPDH expression level. The protein expression levels of the control were set to 1. Data were analyzed using a Student's *t*‐test. Bars and whiskers of the graph represent mean and SD (right panel). (C) Immunoblot analysis was performed to determine TOMM22 and TOMM20 protein levels (*n* = 3). TOMM20, TOMM22, and TOMM40, translocases of outer mitochondrial membrane 20, 22, and 40, respectively (left panel). Protein expressions of TOMM22, TOMM20, TOMM40, and GAPDH were quantified, and normalized by GAPDH expression level. The protein expression levels of the control were set to 1. Data were analyzed using a Student's *t*‐test. Bars and whiskers of the graph represent mean and SD (right panel). (D) Total RNA was isolated from HepG2 cells and subjected to qPCR. mRNA expressions of PCK1, PCK2, HMGCS1, and HMGCS2 were quantified (*n* = 4), and normalized by GAPDH mRNA expression level. The mRNA expression levels of the control were set to 1. Data were analyzed using a Student's *t*‐test. Bars and whiskers of the graph represent mean and SD. (E) Immunoblot analysis was performed to determine PCK1, PCK2, HMGCS1, and HMGCS2 protein levels (*n* = 3). PCK1 and PCK2, phosphoenolpyruvate carboxykinase 1 and 2; HMGCS1 and HMGCS2, 3‐hydroxy‐3‐methylglutaryl‐CoA synthase 1 and 2 (upper panel). Protein expressions of PCK1, PCK2, HMGCS1, HMGCS2, and GAPDH was quantified, and normalized by GAPDH expression level. The protein expression levels of the control were set to 1. Data were analyzed using a Student's *t*‐test. Bars and whiskers of the graph represent mean and SD (lower panel).

When unfolded proteins accumulate in the mitochondria, UPR^mt^‐related transcription factors such as ATF4, ATF5, and DDIT3 are generally upregulated [14]. They induce the expression of several mitochondrial proteases and chaperones, including HSPD1 and LONP1, to maintain mitochondrial quality. To examine whether the depletion of the mitochondrial intermembrane protein FAM136A also induced the UPR^mt^, we measured the protein levels of ATF4, ATF5, DDIT3, LONP1, and HSPD1 (Fig. 3A,B). We observed a significant increase in the expressions of ATF4, DDIT3 (Fig. 3A), and LONP1 (Fig. 3B). We next examined the expression of TOMM22 and TOMM20 (Fig. 3C) because we previously found that depletion of TMEM65 resulted in increased expression of TOMM22 [16] and that depletion of TMEM160 resulted in increased expression of TOMM22 and TOMM20 [17]. Both TOMM22 and TOMM20 were significantly increased, suggesting that protein import into mitochondria was accelerated under UPR^mt^, as previously reported [15]. First, whether any cellular events may prevent the cell from apoptosis induced by increased ROS in FAM136A‐depleted cells was speculated. Therefore, we first hypothesized that the mitochondrial metabolism might also be regulated during oxidative stress to avoid the overproduction of harmful ROS and that the enzymes involved in gluconeogenesis or ketogenesis may contribute to reduced metabolism in the TCA cycle. Indeed, phosphoenolpyruvate carboxykinases (PCK1 and PCK2) have been shown to reduce ROS [22]. Therefore, we analyzed the mRNA and protein expression levels (Fig. 3D,E) of gluconeogenic phosphoenolpyruvate carboxykinases and ketogenic 3‐hydroxy‐3‐methylglutaryl‐CoA synthases (HMGCS1 and HMGCS2) in FAM136A‐depleted cells. Subsequently, FAM136A depletion was observed to upregulate both the mRNA and protein expression levels of PCK1, PCK2, HMGCS1, and HMGCS2. These results appeared to support the idea that each of these four enzymes may play a role in preventing the production of harmful ROS by reducing the metabolism in the TCA cycle.

### PCK1 overexpression strongly suppresses the enhancement of ROS production induced by FAM136A depletion

**Fig. 4:**
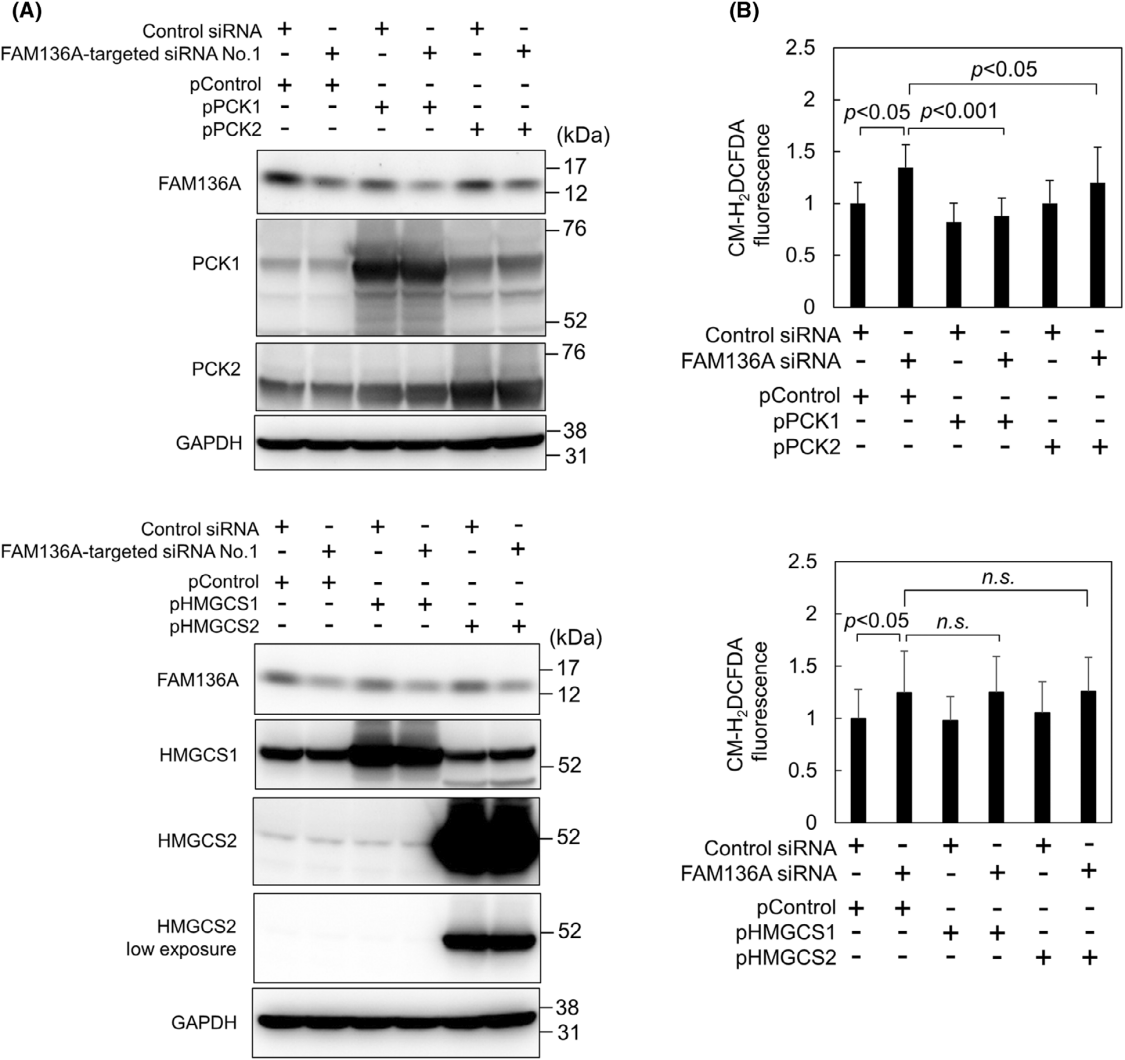
PCK1 overexpression suppresses ROS generation induced by FAM136A depletion. HepG2 cells cotransfected with siRNAs (negative control siRNA or FAM136A‐targeted siRNA No.1) and plasmids (pControl, pPCK1, pPCK2, pHMGCS1 or pHMGCS2), as indicated in the figure. The cells were cultured for 72 h and then subjected to the following analyses: (A) Proteins extracted from the cells were subjected to immunoblot analysis to confirm FAM136A, PCK1, PCK2, HMGCS1, and HMGCS2 protein levels. (B) Fluorescence intensity of CM‐H_2_DCFDA‐loaded cells was measured using Infiniti F200 Pro (*n* = 16). The levels of CM‐H_2_DCFDA fluorescence in the control were set to 1. Data were analyzed using a Student's *t*‐test. Bars and whiskers of the graph represent mean and SD.

To clarify whether PCKs and HMGCSs can prevent the overproduction of ROS indices by FAM136A‐depletion, we performed cotransfection experiments using both siRNAs and plasmids expressing PCK1, PCK2, HMGCS1, and HMGCS2 (Fig. 4). Immunoblot analysis suggested that both the partial depletion of FAM136A and the overexpression of these four enzymes were successful (Fig. 4A). ROS generation was detected using CM‐H_2_DCFDA. ROS generation in cells cotransfected with FAM136A siRNA and the control plasmid was higher than that in cells cotransfected with control siRNA and the control plasmid, suggesting that ROS generation increased even when FAM136A was moderately depleted. In contrast, a strong decrease in ROS generation was observed in cells overexpressing PCK1 whenever FAM136A was depleted, indicating that PCK1 suppresses ROS generation. Moderate suppression of ROS generation was observed in cells overexpressing PCK2.

### FAM136A depletion results in increased expression of cytochrome c proteins, decreased expression of HCCS, and lower ratio of heme/cytochrome c proteins

**Fig. 5:**
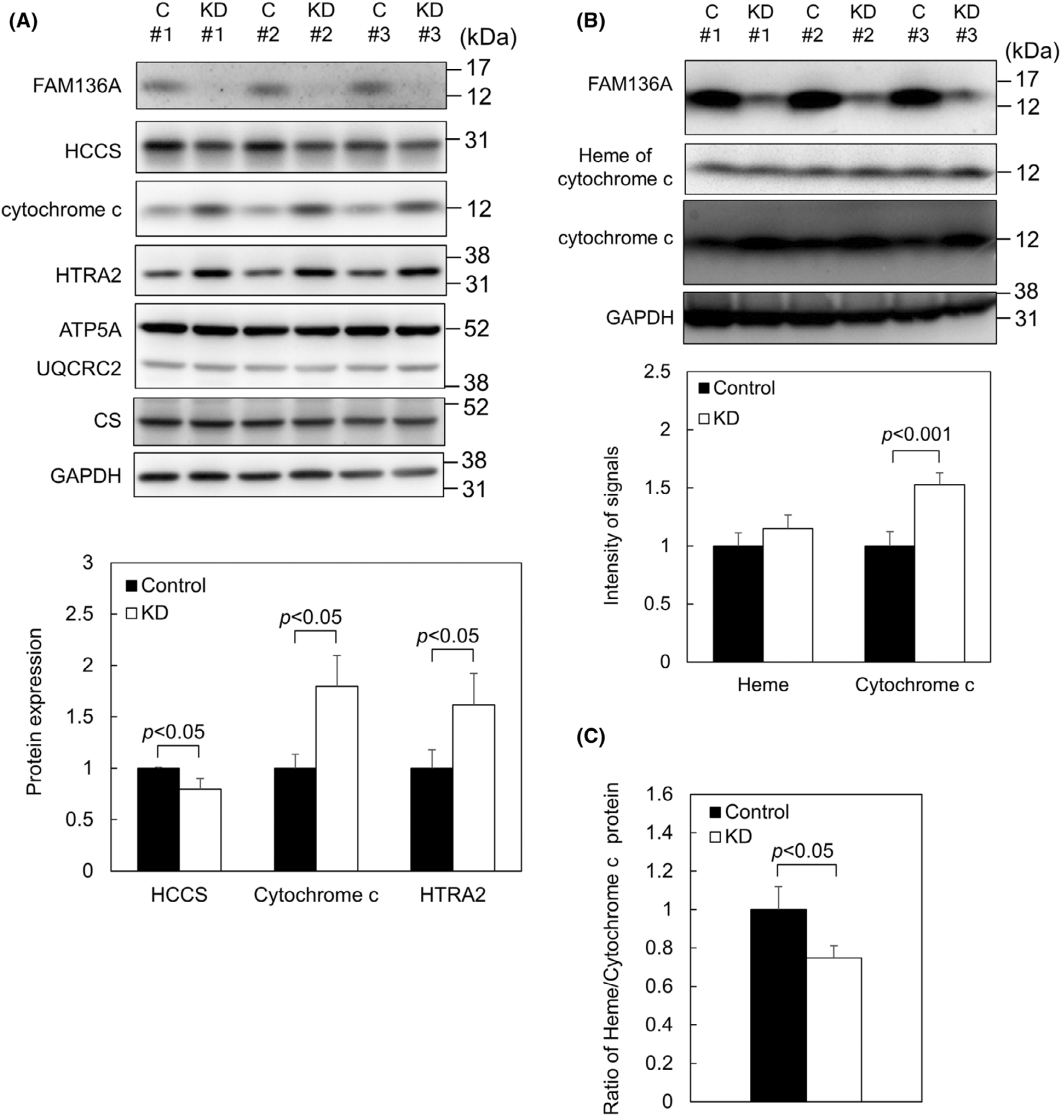
FAM136A depletion results in increased expression of cytochrome c proteins, decreased HCCS expression, and lower ratio of heme/cytochrome c proteins. HepG2 cells were transfected with negative control siRNA or FAM136A‐targeted siRNA No.1 and cultured for 72 h. Then, proteins extracted from the cells were mixed with SDS/PAGE loading buffer without 2‐ME. (A) The samples were subjected to immunoblot analysis to determine HCCS, cytochrome c, and HTRA2 protein levels (*n* = 3). C, control cell lysate; HCCS, holocytochrome c synthase; HTRA2, high temperature requirement protein A2; KD, FAM136A‐depleted cell lysate obtained by using FAM136A‐targeted siRNA No.1 (upper panel). Protein expressions of HCCS, cytochrome c, and HTRA2 were quantified, and normalized by GAPDH expression level. The protein expression levels of the control were set to 1. Data were analyzed using a Student's *t*‐test. Bars and whiskers of the graph represent mean and SD (lower panel). (B) Heme in cytochrome c was stained using ECL, and then followed by immunostaining using anti‐cytochrome c antibody (upper panel). The intensity of the bands corresponding to heme in cytochrome c and cytochrome c proteins was quantified, and normalized by GAPDH expression level. The intensity of the control was set to 1. Data were analyzed using a Student's *t*‐test. Bars and whiskers of the graph represent mean and SD (lower panel). (C) The ratio of heme/cytochrome c protein was calculated. The ratio of the control was set to 1. Data were analyzed using a Student's *t*‐test. Bars and whiskers of the graph represent mean and SD.

Recent interactome analysis has indicated that FAM136A interacts with holocytochrome c synthase (HCCS) [23], a mitochondrial inner membrane‐localized enzyme that faces IMS [28]. HCCS catalyzes the reaction to make covalent bond between heme and apocytochrome c [29, 30]. Therefore, HCCS expression levels were examined next, and FAM136A depletion was found to reduce HCCS expression (Fig. 5A). In addition, expressions of both cytochrome c and HTRA2 were increased. Based on this, we hypothesized that increased HTRA2 in FAM136A‐depleted cells might be necessary to digest increased apocytochrome c. Therefore, we next performed an experiment to quantify the amount of heme in cytochrome c and cytochrome c protein. To determine the amount of heme in cytochrome c protein, we prepared samples using SDS/PAGE loading buffer without a reducing agent 2‐ME to maintain the covalent bond between heme and cytochrome c protein. Heme staining was performed using ECL on PVDF membrane, and cytochrome c proteins were subsequently detected using the same membrane (Fig. 5B). As a result, the amount of heme in holocytochrome c did not change due to FAM136A depletion. However, the amount of cytochrome c protein significantly increased. When the signal intensity of heme was normalized by that of cytochrome c protein, a significant decrease in the ratio of heme to cytochrome c protein was observed (Fig. 5C), suggesting that heme‐unbound apocytochrome c increases in FAM136A‐depleted cells.

### HepG2 cellular morphology did not change from depletion of FAM136A depletion

**Fig. 6:**
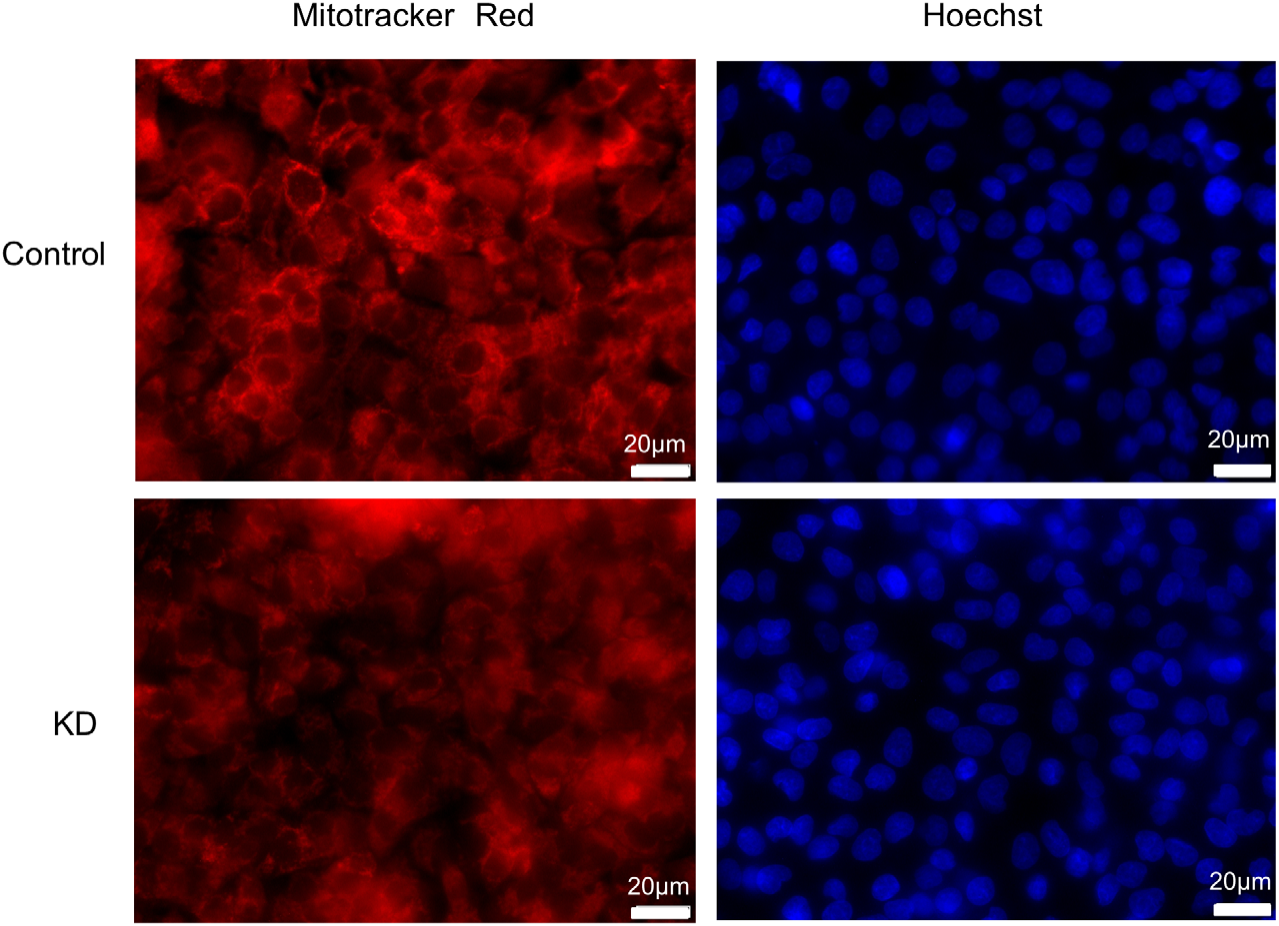
Morphology of HepG2 cells did not change by depletion of FAM136A. HepG2 cells transfected with negative control siRNA or FAM136A‐targeted siRNA No.1 were cultured for 72 h, and stained by Mito Tracker Red and Hoechst dye. After fixation, observing multiple areas of the slide glass under a fluorescent microscope, and confirming that there are no morphological changes, representative cell images were photographed.

To analyze whether FAM136A depletion causes morphological changes in HepG2 cells, we observed the morphology of FAM136A‐depleted cells using Mito Tracker Red (Fig. 6). However, no evident morphological changes could be observed. The mitochondria stained using Mito Tracker Red in FAM136A‐depleted cells were darker than those in control cells. This observation is attributable to the characteristics of Mito Tracker Red that give red fluorescence depending on mitochondrial membrane potential. Thus, these observations also suggest that the membrane potential of mitochondria in FAM136A‐depleted cells is lower than that of mitochondria in the control cells (Fig. 2C).

### Experiments using FAM136A‐targeted siRNA No.2 gave similar results as those using siRNA No.1

**Fig. 7:**
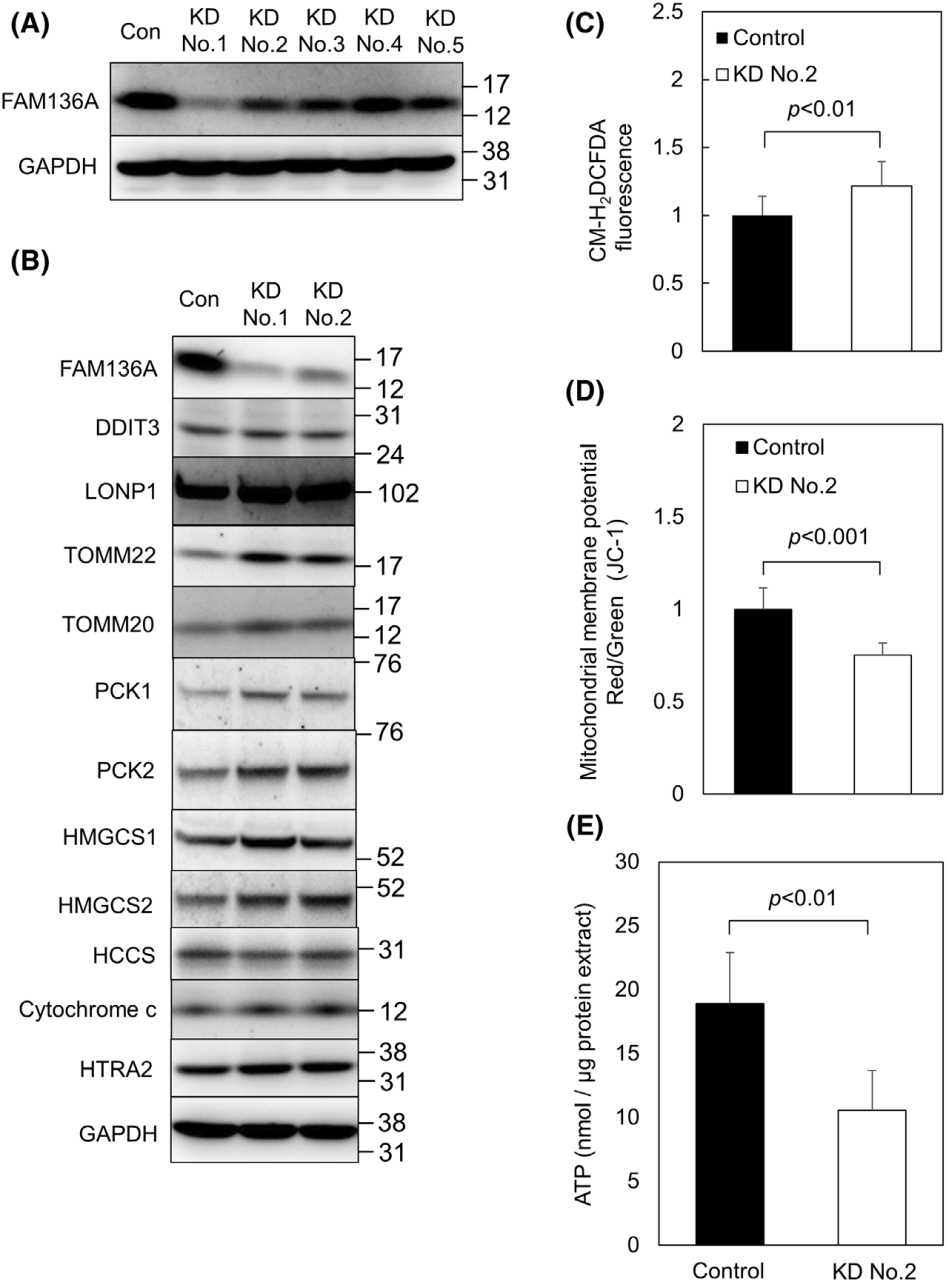
Experiments using FAM136A‐targeted siRNA No.2 gave similar results as those using the FAM136A‐targetted siRNA No.1. HepG2 cells were transfected with negative control siRNA or FAM136A‐targeted siRNAs and cultured for 72 h. (A) Proteins extracted from the cells transfected with negative control siRNA and FAM136A‐targeted siRNAs No.1–5 were subjected to immunoblot analysis to search FAM136A knockdown levels. Con, control cell lysate; KD No.1–5, FAM136A‐depleted cell lysate obtained by using FAM136A‐targeted siRNA No.1–5. (B) Proteins extracted from the cells transfected with negative control siRNA and FAM136A‐targeted siRNAs No.1 and 2 were subjected to immunoblot analysis. (C) Fluorescence intensity of CM‐H_2_DCFDA‐loaded cells transfected with negative control siRNA (Control) and FAM136A‐targeted siRNAs No.2. (KD No.2) was measured using Infiniti F200 Pro (*n* = 8). The levels of CM‐H_2_DCFDA fluorescence in the control were set to 1. Data were analyzed using a Student's *t*‐test. Bars and whiskers of the graph represent mean and SD. (D) Fluorescence intensity of JC‐1‐loaded cells transfected with negative control siRNA (Control) and FAM136A‐targeted siRNAs No.2. (KD No.2) was measured using Infiniti F200 Pro (*n* = 16). The ratio of red fluorescence to green fluorescence (Red/Green) was calculated. The ratio of the control was set to 1. Data were analyzed using a Student's *t*‐test. Bars and whiskers of the graph represent mean and SD. (E) ATP content in the cells transfected with negative control siRNA (Control) and FAM136A‐targeted siRNAs No.2. (KD No.2) was quantified, and normalized by protein concentration (*n* = 4). Data were analyzed using a Student's *t*‐test. Bars and whiskers of the graph represent mean and SD.

To confirm that the results obtained by using FAM136A‐targeted siRNA No.1 were due to the depletion of FAM136A, we performed several additional experiments using other FAM136A‐targeted siRNAs (Fig. 7). First, we examined the knockdown efficiency of FAM136A‐targeted siRNA No.1‐5 (Fig. 7A). Unfortunately, the siRNAs No. 2–5 showed lower knockdown efficiency than FAM136A‐targeted siRNA No.1. Therefore, we reluctantly performed further experiments using FAM136A‐targeted siRNA No.2. When the FAM136A‐targeted siRNA No.2 was transfected into HepG2 cells, we observed similar or lower changes in protein expression levels as is observed in the cells transfected with FAM136A‐targeted siRNA No.1 (Fig. 7B). Furthermore, we tested the effects of FAM136A‐targeted siRNA No.2 on ROS production level, mitochondrial membrane potential, and ATP production (Fig. 7C–E). When the FAM136A‐targeted siRNA No.2 was used, we observed a significant increase in the ROS level (Fig. 7C), a significant decrease in mitochondrial membrane potential (Fig. 7D), and a significant decrease in ATP content (Fig. 7E). However, the extent of these changes was lower than when the FAM136A‐targeted siRNA No.1 was used (Fig. 2B–D).

## Discussion

**Fig. 8:**
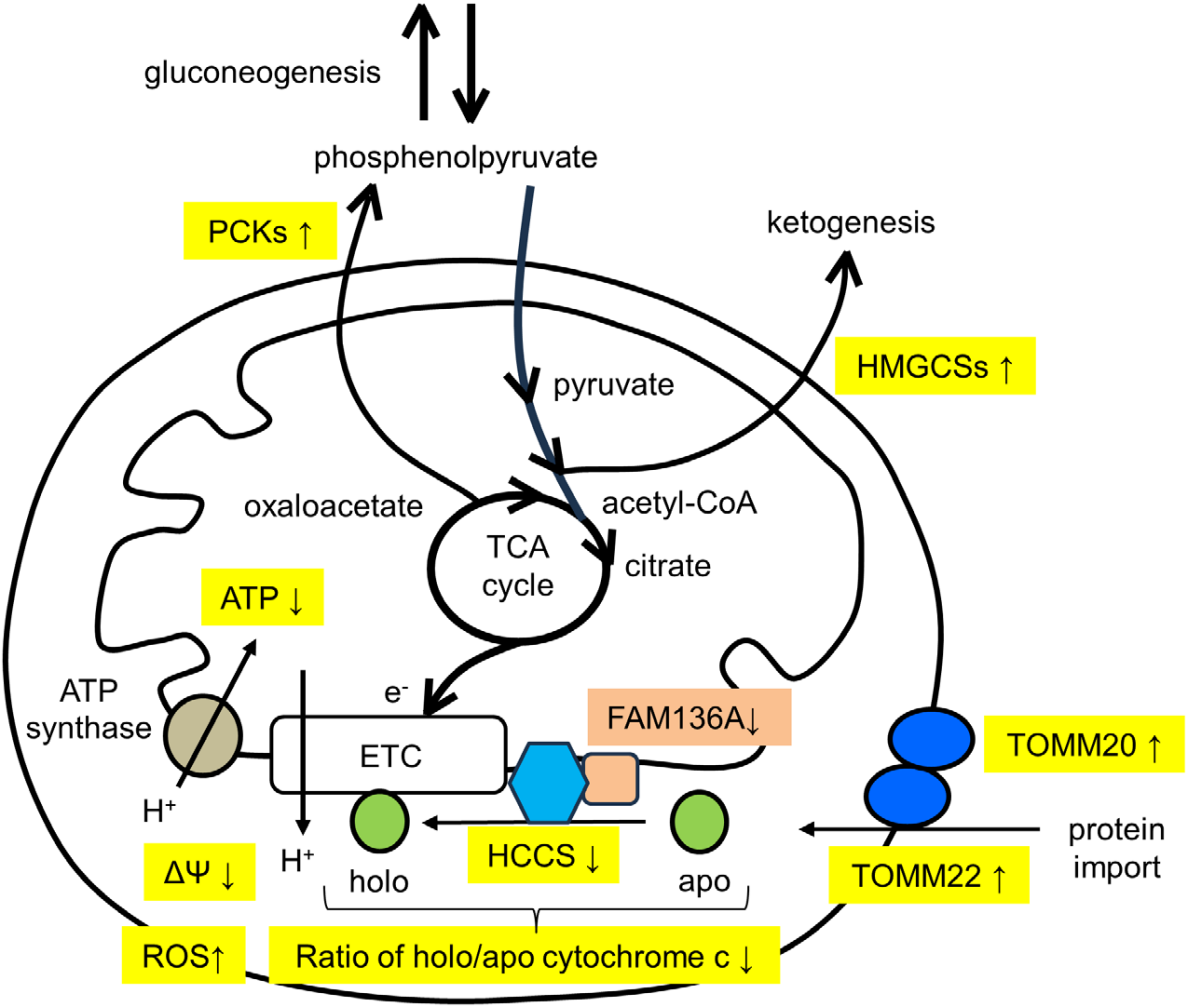
Summary of the phenotypes observed in FAM136A‐depleted cells. FAM136A depletion upregulated ROS production, reduced mitochondrial membrane potential (ΔΨ) and ATP production, and upregulated expression of the four enzymes (PCK1, PCK2, HMGCS1, and HMGCS2) that would reduce metabolisms in TCA cycle. The expression of both TOMM22 and TOMM20 that would probably enhance mitochondrial protein import was also upregulated. FAM136A depletion reduced expression level of FAM136A‐interacting protein HCCS that produce holocytochrome c by combining heme to apocytochrome c. In FAM136A‐depleted cells, the amount of heme bound to cytochrome c was not affected although the expression level of cytochrome c was significantly upregulated, indicating that the ratio of holocytochrome c/apocytochrome c is reduced.

Several studies have suggested that *FAM136A* is a candidate gene for Ménière's disease [19]. However, the exact function of FAM136A remains unclear. A recent analysis using *C. elegans* indicated that the loss of FAM136A function results in minor but significant changes in locomotion and behavior [20], so we hypothesized that FAM136A may play a fundamental role in every cell type. Therefore, we analyzed this protein in HepG2 cells to elucidate its function. Prior to the analysis, we confirmed the localization of this protein as only one report had suggested localization of FAM136A in the mitochondrial IMS [18]. FAM136A localization in the mitochondrial IMS was confirmed based on our analysis. As no other functional information on this protein has been reported thus far, we analyzed the influence of FAM136A depletion on HepG2 cells using siRNA. We found some phenotypes that may provide clues to understanding the function of FAM136A (Fig. 8). FAM136A depletion induced ROS production, indicating that FAM136A depletion induces oxidative stress. Mild upregulation of ATF4, DDIT3, and LONP1 was observed suggesting that UPR^mt^ was evoked to recover mitochondrial damage by ROS. Additionally, these observations are attributable to integrated stress response (ISR) [31]. Both mitochondrial membrane potential and ATP were reduced upon the depletion. Therefore, we examined whether CASP9 cleavage was enhanced, but no significant increases in cleavage were observed. Subsequently, cellular responses were hypothesized to regulate ROS production. ROS production is controlled by the regulation of metabolism during the TCA cycle. Interestingly, we found that the expressions of enzymes involved in gluconeogenesis (PCK1 and PCK2) and ketogenesis (HMGCS1 and HMGCS2) were upregulated by FAM136A depletion. In addition, we demonstrated that overexpression of PCK1 significantly reduced the overproduction of ROS induced by FAM136A depletion. This result indicates that PCK1 suppressed ROS generation, which was also recently reported [22]. Recent analyses have revealed that PCK1, PCK2, HMGCS1, and HMGCS2 are important for regulating and maintaining mitochondrial function. Verissimo *et al*. showed that PCK1 is a key regulator of metabolic and mitochondrial functions in renal tubular cells [32]. Bluemel *et al*. demonstrated that PCK2 regulates mitochondrial respiration and maintains the redox balance in nutrient‐deprived human lung cancer cells [33]. Zhou *et al*. suggested that HMGCS1 upregulates UPR downstream components and protects the mitochondria and endoplasmic reticulum from damage in acute myeloid leukemia cells under stress [34]. Arima *et al*. showed that ketogenesis mediated by HMGCS2 plays a protective role in mitochondrial function [35]. Finally, given the suggested interaction between FAM136A and HCCS based on interactome analysis [23], we examined the expression level of HCCS. We found reduced expression of HCCS and increases in cytochrome c in FAM136A‐depleted cells. This observation is noteworthy because reducing HCCS may reduce the production of holocytochrome c. Indeed, in FAM136A‐depleted cells, the amount of heme bound to cytochrome c was not significantly different. However, the expression level of cytochrome c was significantly upregulated, indicating that the ratio of holocytochrome c to apocytochrome c is reduced. An increase in apocytochrome c may inhibit electron transport in ETC, reduce membrane potential, inhibit ATP production, reduce metabolism in the TCA cycle, and enhance upregulation of the enzymes that reduce substrates in the TCA cycle, such as PCKs and HMGCSs, as is observed in FAM136A‐depleted cells. The formation of apoptosome during apoptosis and cleavage of CASP9 may be incomplete when the ratio of holocytochrome c to apocytochrome c is reduced. The low levels of cleaved CASP9 observed in FAM136A‐depleted cells may also be due to excessive accumulation of apocytochrome c. The impairment of HCCS activates a noncanonical cell death pathway [36]. The observed upregulation of HTRA2 in FAM136A‐depleted cells may be necessary to remove excessively accumulated apocytochrome c.

## Conclusions

Although further investigation is necessary to reveal the role of FAM136A, we have shown that depletion of FAM136A leads to ROS overproduction, a decrease in mitochondrial membrane potential, decrease of ATP production, induction of the UPR^mt^, and an increase in the enzymes involved in gluconeogenesis (PCK1 and PCK2) and ketogenesis (HMGCS1 and HMGCS2). We demonstrated that overexpression of PCK1 suppressed ROS generation induced by FAM136A depletion. Notably, we have shown that depletion of FAM136A leads to reduced HCCS levels, a FAM136A‐interacting enzyme, and the amount of heme in holocytochrome c did not increase significantly. However, the amount of cytochrome c proteins increased significantly, indicating that the synthesis of holocytochrome c by HCCS is reduced in FAM136A‐depleted cells.

## Conflict of interest

The authors declare that they have no conflicts of interest regarding the content of this article.

### Peer review

The peer review history for this article is available at https://www.webofscience.com/api/gateway/wos/peer‐review/10.1002/2211‐5463.13967.

## Author contributions

YO performed experiments. MY performed experiments, analyzed data, and wrote this article.

## Data Availability

The data that support the findings of this study are available from the corresponding author (yano@kumamoto-hsu.ac.jp) upon reasonable request.
